# Intestinal Tuberculosis: A Diagnostic Challenge

**DOI:** 10.7759/cureus.13058

**Published:** 2021-02-01

**Authors:** Hansang Park, Tikal Kansara, Ana M Victoria, Noella Boma, Jungrak Hong

**Affiliations:** 1 Internal Medicine, New York City Health and Hospitals Corporation (NYC HHC) Metropolitan, New York, USA; 2 Internal Medicine, New York Medical College, Metropolitan Hospital Center, New York, USA; 3 Internal Medicine, Metropolitan Hospital, New York, USA

**Keywords:** intestinal tuberculosis, tuberculosis, mac, mtb, tb, mycobacterium tuberculosis, mycobacterium, abdominal tb, abdominal tuberculosis, intestinal tb

## Abstract

Diagnosing intestinal tuberculosis (TB) with uncommon clinical manifestations is often challenging. Here, we report a case of an alcoholic patient who presented with vague symptoms and was later diagnosed with intestinal TB. This patient experienced multiorgan failure causing hemodynamic instability requiring ionotropic support; acute hypoxic respiratory failure managed with non-invasive positive pressure ventilation, hepatic failure, transudative peritoneal effusion, and transudative pleural effusion. These conditions clouded our judgment to pursue colonoscopy for a definite diagnosis and delayed the anti-tuberculosis treatment. When intestinal tuberculosis TB is suspected, the differential diagnosis must be established with other gastrointestinal involving diseases, including mycobacterium avium complex (MAC) and Crohn's disease (CD). MAC can show overlapping features with intestinal TB or coexist with it; Acid-fast stain and tissue culture are the key tests to differentiate these two. In the presence of diagnostic uncertainty between intestinal TB and CD, a therapeutic trial with anti-tuberculous therapy may be warranted.

## Introduction

Intestinal tuberculosis is an uncommon clinical manifestation of tuberculosis, representing approximately 5% of extra-pulmonary cases reported in the US [1-3]. Possible pathophysiology includes swallowing of sputum with direct seeding, hematogenous spread, or ingestion of milk from cows affected by bovine TB, which mainly occurs in developing nations [4]. The most commonly affected parts of the intestine are the terminal ileum and cecum. Typical manifestations are abdominal pain, intestinal swelling, intestinal obstruction, hematochezia, a palpable abdominal mass, fever, weight loss, and night sweats [5]. The differentiation between Crohn's disease (CD) and intestinal tuberculosis would be difficult since there is a substantial overlap between its features, especially when a case like this one does not have the clear risk factors of tuberculosis [6]. The treatment approach for intestinal tuberculosis is the same as for pulmonary TB in general [7].

## Case presentation

A 37-year-old Mexican male with a history of heavy alcohol abuse, drinking more than 12 standard drinks/day for more than 17 years, presented to the emergency room (ER) complaining of a two-week history of progressive intermittent diffuse abdominal pain, unintentional weight loss, and chronic intermittent non-bloody diarrhea. As per the patient, he moved to the United States when he was 17 years old.

**Figure 1:**
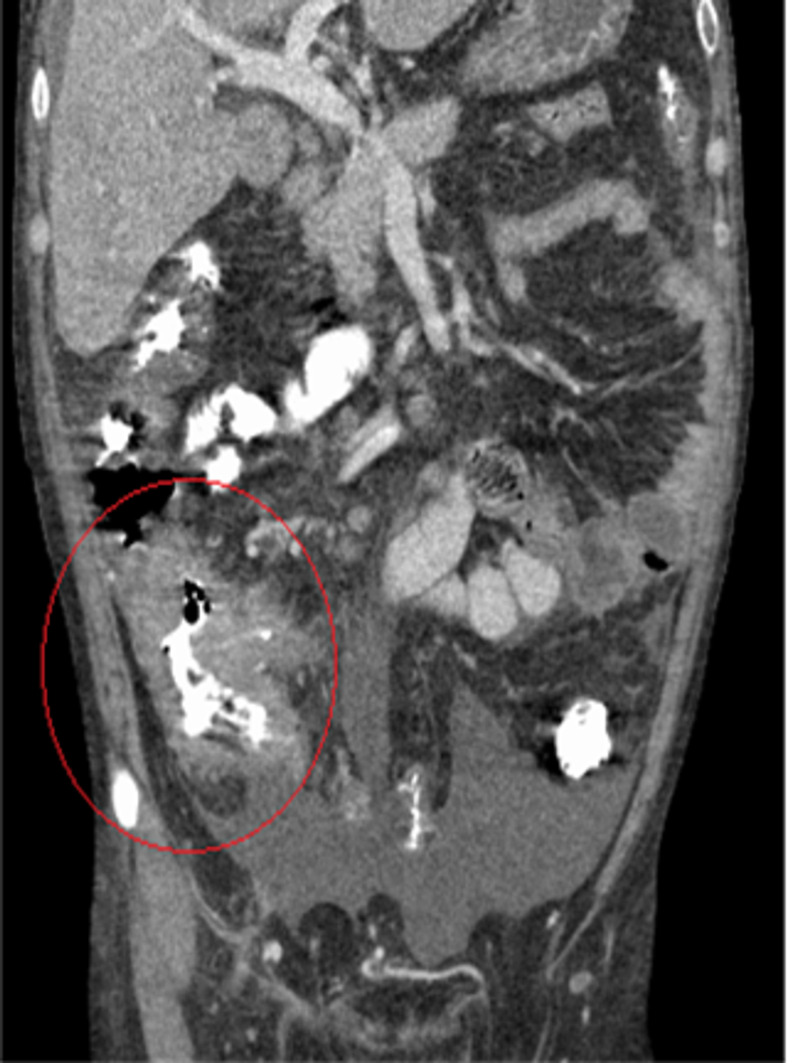
Thickening of the terminal ileum and cecum

Upon arrival to ER, he was found to be hypotensive: 92/50 millimeter mercury, tachycardic: 105 per minute, and afebrile. Physical examination revealed diffuse abdominal tenderness and abdominal distention with shifting dullness. His laboratory investigations showed a total leukocyte count of 14.18 cells per cubic millimeter with neutrophilia of 79% and normocytic normochromic anemia of hemoglobin 7.1 gram per deciliter. Prothrombin time (PT)/international normalized ratio (INR) was 17.5 seconds/1.5, respectively. The hepatic function test showed aspartate transaminase 76 units per liter, alanine transaminase 21 units per liter, alkaline phosphatase 457 units per liter, albumin 1.6 gram per deciliter, and gamma-glutamyl transferase 502 international units per liter. Human immunodeficiency virus screening, carcinoembryonic antigen, alpha-fetoprotein, and gastrointestinal pathogen stool polymerase chain reaction (PCR) panel were all negative. CT abdomen and pelvis with oral and intravenous (IV) contrast revealed thickening of the terminal ileum and cecum, to a lesser extent to the ascending and transverse colon (Figure 1). Ascites, fatty liver, and right lower quadrant lymphadenopathy were also pertinent positive findings.

Based on the patient's clinical presentation, laboratory investigations, and CT findings, our initial differential diagnoses were inflammatory bowel disease, malignancy (adenocarcinoma, lymphoma, other), and infectious colitis.

The patient was started with broad-spectrum intravenous antibiotics while waiting for other test results for infectious causes and inflammatory diseases, including cultures, PCRs, inflammatory markers, and autoantibodies, to come back. After admission, the patient slowly deteriorated as his ascites and pleural effusion progressed, causing worsening of dyspnea followed by hypoxic respiratory failure secondary to the mass effect of ascites. The patient did not require intensive care unit stay or inotropes; requiring IV fluids resuscitation and saturation maintained on non-invasive positive pressure ventilation. In light of unresponsiveness to broad-spectrum antibiotics, autoimmune diseases were considered and intravenous steroids were given as an empirical trial, which showed no clinical improvement. Paracentesis and thoracentesis were done to characterize the fluid. The pleural and ascitic fluid analysis was consistent with transudate in view of low cell count, low lactate dehydrogenase (LDH), and serum ascites albumin gradient (SAAG) less than 1.1 gram per deciliter. Colonoscopy findings were ileitis and severe diffuse inflammation in the cecum, ascending colon, and proximal transverse colon. Finally, it was after the colonoscopy-guided biopsy, which revealed acid-fast stain bacilli, that we decided to start anti-tuberculosis treatment with a standard four-drug regimen, including rifampin, isoniazid, pyrazinamide, and ethambutol. The patient started to improve and was discharged after five days with outpatient follow-up. The patient's acid-fast bacilli (AFB) culture from sputum and stool both grew mycobacterium tuberculosis and mycobacterium avium complex. When the patient returned to the clinic four weeks after the discharge, he reported resolution of his symptoms. His blood tests, including complete blood count (CBC) and comprehensive metabolic panel (CMP) were all found to be normal.

## Discussion

Intestinal TB should always be considered when dealing with a patient with B-symptoms, abdominal pain, abdominal distention, ascites, hepatomegaly, diarrhea, and abnormal liver function tests. Intestinal TB and CD share many clinical features, making differential diagnoses always challenging, especially if they do not have any risk factors for TB infection. Differential points are the presence of ascites, both-sides of ileocecal valve involvement leading to incompetence, submucosal large caseation granuloma, all of which are uncommon in CD [6,8-13]. Identifying TB in a timely manner is crucial because of its high mortality, ranging from 8% to 50% in many studies. Factors related to poor prognosis in intestinal TB include the patient's age, pre-existing liver cirrhosis, and delay in initiating anti-tuberculosis treatment [5,14-16].

Clinical deterioration in intestinal TB is often caused by using immunosuppressive medications secondary to misdiagnosis. In our case, the patient’s clinical condition deteriorated due to a delay in anti-tuberculosis treatment and a trial of steroids. Later, both MAC and mycobacterium tuberculosis (MTB) were cultured in the patient's stool. But in sputum culture, only MAC was detected without MTB; a possible explanation for this would be the low sensitivity of the culture. Compared to sputum smears, sputum cultures have a much higher sensitivity, but it is still not high enough (only 82% in a large study). This means that sputum cultures are not positive in every person with TB, and people can have TB even if the sputum culture results come back negative.

Anti-tuberculosis therapy is the treatment of choice, but in the case of perforation, fistula, bleeding, obstruction, and abscess, patients might need surgical interventions as well [17]. The most common complication in untreated intestinal TB is intestinal obstruction secondary to prolonged stricture [18]. The selection of the anti-tuberculosis therapy regimen is identical to pulmonary TB treatment in general [5,7,19].

## Conclusions

Determining the etiology of ileitis, or inflammation of the ileum is often challenging due to many medical diseases involving the ileum, including infections and systemic disorders, Crohn's disease, intestinal TB, bacterial infection, vasculitides, ischemia, neoplasms, spondyloarthropathies, and medication-induced or eosinophilic enteritis. The diagnosis of ileitis is of paramount importance because misdiagnosis or delayed diagnosis may result in critical management errors. Given the clinical, endoscopic, and histopathological similarities of ileocolic Crohn's disease and intestinal TB, acid-fast stain and tissue culture could be the critical tests to differentiate these two conditions. No clear guideline exists to distinguish various forms of ileitis, and it remains a test of clinical acumen. Nonetheless, a therapeutic trial with anti-tuberculosis therapy may warrant diagnostic uncertainty.

## References

[1] (2017). Centers for Disease Control and Prevention. Reported tuberculosis in the United States. https://www.cdc.gov/tb/statistics/reports/2017/2017_Surveillance_FullReport.pdf.

[2] Rathi P, Gambhire P (2016). Abdominal tuberculosis. J Assoc Physicians India.

[3] Evans RP, Mourad MM, Dvorkin L, Bramhall SR (2016). Hepatic and intra-abdominal tuberculosis: 2016 update. Curr Infect Dis Rep.

[4] Al-Quorain AA, Facharzt Facharzt, Satti MB, Al-Freihi HM, Al-Gindan YM, Al-Awad N (1993). Abdominal tuberculosis in Saudi Arabia: a clinicopathological study of 65 cases. Am J Gastroenterol.

[5] Debi U, Ravisankar V, Prasad KK, Sinha SK, Sharma AK (2014). Abdominal tuberculosis of the gastrointestinal tract: revisited. World J Gastroenterol.

[6] Almadi MA, Ghosh S, Aljebreen AM (2009). Differentiating intestinal tuberculosis from Crohn's disease: a diagnostic challenge. Am J Gastroenterol.

[7] Jullien S, Jain S, Ryan H, Ahuja V (2016). Six-month therapy for abdominal tuberculosis. Cochrane Database Syst Rev.

[8] Pratap Mouli V, Munot K, Ananthakrishnan A (2017). Endoscopic and clinical responses to anti-tubercular therapy can differentiate intestinal tuberculosis from Crohn's disease. Aliment Pharmacol Ther.

[9] Kedia S, Sharma R, Sreenivas V (2017). Accuracy of computed tomographic features in differentiating intestinal tuberculosis from Crohn's disease: a systematic review with meta-analysis. Intest Res.

[10] Makharia GK, Srivastava S, Das P (2010). Clinical, endoscopic, and histological differentiations between Crohn's disease and intestinal tuberculosis. Am J Gastroenterol.

[11] Li X, Liu X, Zou Y (2011). Predictors of clinical and endoscopic findings in differentiating Crohn's disease from intestinal tuberculosis. Dig Dis Sci.

[12] Yu H, Liu Y, Wang Y, Peng L, Li A, Zhang Y (2012). Clinical, endoscopic and histological differentiations between Crohn's disease and intestinal tuberculosis. Digestion.

[13] Bae JH, Park SH, Ye BD (2017). Development and validation of a novel prediction model for differential diagnosis between Crohn's disease and intestinal tuberculosis. Inflamm Bowel Dis.

[14] Aguado JM, Pons F, Casafont F, San Miguel G, Valle R (1990). Tuberculous peritonitis: a study comparing cirrhotic and noncirrhotic patients. J Clin Gastroenterol.

[15] Chow KM, Chow VC, Hung LC, Wong SM, Szeto CC (2002). Tuberculous peritonitis-associated mortality is high among patients waiting for the results of mycobacterial cultures of ascitic fluid samples. Clin Infect Dis.

[16] Wang HK, Hsueh PR, Hung CC, Chang SC, Luh KT, Hsieh WC (1998). Tuberculous peritonitis: analysis of 35 cases. J Microbiol Immunol Infect.

[17] Kapoor VK (1998). Abdominal tuberculosis. Postgrad Med J.

[18] Ha HK, Ko GY, Yu ES (1999). Intestinal tuberculosis with abdominal complications: radiologic and pathologic features. Abdom Imaging.

[19] Makharia GK, Ghoshal UC, Ramakrishna BS (2015). Intermittent directly observed therapy for abdominal tuberculosis: a multicenter randomized controlled trial comparing 6 months versus 9 months of therapy. Clin Infect Dis.

